# Comparative Analysis of Heat Exposure‐Induced Molecular Changes in Two Turtle Species with Contrasting Thermal Adaptations

**DOI:** 10.1111/1749-4877.13011

**Published:** 2025-07-17

**Authors:** Jian Hong, Yangchun Gao, Jiaxuan Li, Yan Ge, Yufeng Wei, Youqiang Yin, Qianru Liang, Shiping Gong

**Affiliations:** ^1^ College of Life Science and Technology Jinan University Guangzhou China; ^2^ Guangdong Key Laboratory of Animal Conservation and Resource Utilization, Institute of Zoology Guangdong Academy of Sciences Guangzhou China

**Keywords:** endoplasmic reticulum, heat stress, *Platysternon megacephalum*, *Trachemys scripta elegans*, transcriptomic analysis

## Abstract

Global climate change has heightened heat stress, threatening amphibian and reptile survival, including turtles. Although turtle species vary in heat tolerance, the molecular mechanisms behind these differences are not well understood. This study aimed to identify differentially expressed genes (DEGs) in response to heat stress (32°C) versus normal temperature (25°C) in eight tissues (brain, heart, intestine, liver, lung, muscle, spleen, and stomach) of two turtle species: *Platysternon megacephalum* (low heat tolerance) and *Trachemys scripta elegans* (high heat tolerance) using RNA‐seq. The results revealed significant down‐regulation of genes involved in energy and lipid metabolism in *P. megacephalum*, suggesting metabolic suppression under heat stress. Furthermore, the jumonji and AT‐rich interaction domain containing 2 (JARID2) gene, which regulates cell proliferation and differentiation, was up‐regulated in all tissues of *P. megacephalum* but down‐regulated in all tissues of *T. scripta elegans* under heat stress. Pathway analysis revealed that protein processing in the endoplasmic reticulum was significantly enriched in brain, heart, lung, and muscle tissues of *P. megacephalum*, with *BiP, CHOP*, *NEF*, and *HSPs* significantly up‐regulated in brain tissue, highlighting this pathway's impact on heat stress response. Seven hub genes were identified in the protein processing in the endoplasmic reticulum pathway in *P. megacephalum*. In contrast, *T. scripta elegans* showed a moderate response, with up‐regulation of ribosomal genes in the brain to enhance protein synthesis and folding, while down‐regulation of cell cycle genes in the intestine helped conserve energy for cellular repair. No significant pathways were found in other tissues of *T. scripta elegans*. These molecular responses in *T. scripta elegans* likely contribute to its better adaptation to heat stress. This study provides new insights into the molecular mechanisms of heat stress adaptation in turtles, offering valuable knowledge for understanding their ability to cope with future climate change.

## Graphical abstract

Protein processing in the endoplasmic reticulum is highlighted in response to heat stress in *Platysternon megacephalum*. Under heat stress, the up‐regulation of genes such as *CHOP* in protein processing in the endoplasmic reticulum pathway, along with the suppression of energy and lipid metabolism and the up‐regulation of *JARID2* expression, leads to physiological dysfunction and reduces heat tolerance in *P. megacephalum*. In contrast, under heat stress, *Trachemys scripta elegans* enhances its heat tolerance by up‐regulating ribosome pathway genes and down‐regulating cell cycle pathway genes, as well as *JARID2* expression.

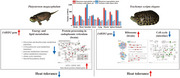

## 1 Introduction

Global climate change poses a major threat to biodiversity (Garcia et al. 2014; Barbarossa et al. 2021). It has increased the frequency and intensity of heatwaves (Perkins‐Kirkpatrick and Lewis 2020), significantly affecting the behavior, growth, embryonic development, and physiological processes of ectothermic animals (Jørgensen et al. 2022), often leading to mass mortality or local extinction (Ma et al. 2021; Bhagarathi et al. 2024). Turtles, a highly endangered ectothermic group (Stanford et al. 2020), are particularly vulnerable to heat. Negative effects of high temperatures include elevated metabolism, compromised immunity, reduced reproduction, and mortality (Patrício et al. 2021; Jiang et al. 2024; Gao et al. 2021). However, tolerance to heat stress varies across turtle species. For example, *Trachemys scripta elegans* has an upper thermal limit of 32°C for normal growth (Burger 2009; Dang et al. 2019), while *Mauremys mutica* and *Mauremys reevesii* have limits of 30°C and 32°C, respectively (Lu et al. 2020; Wei et al. 2021). In contrast, *Graptemys oculifera* and *Graptemys flavimaculata* have upper limits of 28°C (Jones 1996, 2017), and *Platysternon megacephalum* has a limit of 27°C (Shen et al. 2013; Zhang et al. 2009), indicating higher sensitivity to heat. These species‐specific differences in heat tolerance will influence their ability to cope with climate change (Zhang et al. 2023). However, the molecular mechanisms behind these variations remain poorly understood, limiting our understanding of their potential for adaptation.

Studies on ectothermic animals have primarily focused on groups like fish, crustaceans, and lizards (Bowden et al. 2007; Kazmi et al. 2022; Scudiero et al. 2021). These studies show that heat stress significantly up‐regulates heat shock proteins (HSPs) and molecular chaperones, which are essential for maintaining protein stability and enhancing heat tolerance (Chen et al. 2023; Jeyachandran et al. 2023). For instance, HSP40, HSP90, and HSPA8 have been shown to regulate thermal tolerance in species like *Exopalaemon carinicauda* and *Eremias argus* (Chang et al. 2022; Shi et al. 2020). Additionally, pathways such as protein processing in the endoplasmic reticulum, lipid metabolism, and amino acid metabolism are critical for heat stress adaptation (Zhao et al. 2022). Heat stress often induces ER stress, impacting protein folding and processing, which in turn promotes the expression of protein‐folding chaperones (Schröder 2008). The reorganization of lipid metabolism helps to maintain the fluidity of cellular membranes (Fabri et al. 2020). For example, protein processing in the endoplasmic reticulum in the gills of *Alosa sapidissima*, as well as protein processing in the endoplasmic reticulum and fatty acid metabolism in the liver of *Salmo salar*, is critical for heat stress tolerance (Luo et al. 2024; Shi et al. 2019). Similarly, glucose, lipid, and amino acid metabolism in the liver of *Gymnocypris chilianensis* is crucial for heat stress tolerance (Zhao et al. 2022). These metabolic shifts highlight their role in maintaining homeostasis and enhancing heat resistance. In contrast, studies on molecular responses to heat stress in turtles are limited, often focusing on single species or specific genes. For example, in *M. mutica*, heat stress causes down‐regulation of complement and coagulation cascade genes, suppressing immune and clotting responses (Gao et al. 2021). In *T. scripta elegans*, up‐regulation of phosphoenolpyruvate carboxykinase suggests activation of gluconeogenesis to maintain energy balance under stress (Jiang et al. 2024). In *Caretta caretta* embryos, differential expression of HSPs highlights their role in protein folding and protection against heat‐induced damage (Bentley et al. 2017). However, the molecular mechanisms behind interspecific differences in heat tolerance among turtles remain poorly understood.

Transcriptomic sequencing is a powerful tool for investigating molecular responses to heat stress in ectothermic animals and has been applied to fish (Zhao et al. 2022; Luo et al. 2024), crustaceans (Shi et al. 2020; Zheng et al. 2019), and lizards (Chang et al. 2022; Akashi et al. 2016). Previous studies show that different tissues and organs in ectotherms respond differently to heat stress. For example, in *Hoplosternum littorale*, the liver is more heat‐sensitive than the brain, showing changes in antioxidant enzyme activity and lipid peroxidation, while the brain does not (Rossi et al. 2017). In *Squalius carolitertii*, the liver is more heat‐sensitive than the muscle, while in *Squalius torgalensis*, the skeletal muscle is more sensitive than the liver (Jesus et al. 2016). Similarly, the brain, liver, muscle, and digestive tract of *Amphiprion ocellaris* are all heat‐sensitive (Moore et al. 2024). Therefore, transcriptomics of multiple tissues can help identify which are most vulnerable to heat stress, providing insight into the limitations of adaptation to high temperatures.

To investigate the molecular mechanisms behind differences in heat tolerance among turtles, we selected *P. megacephalum* (low heat tolerance) and *T. scripta elegans* (relatively high heat tolerance). This contrast in heat tolerance provides an ideal framework for exploring how different species adapt to heat stress. Given its restricted distribution and conservation concerns, *P. megacephalum* offers insights into species at the edge of their thermal tolerance (Sung et al. 2013), while *T. scripta elegans*, a more widely distributed species, serves as a comparison due to its greater resilience to heat stress (Jiang et al. 2024). This contrast suggests that *T. scripta elegans* may have evolved specific molecular adaptations enhancing its heat tolerance. By comparing these species, we aimed to identify key genes and metabolic pathways contributing to their differing abilities to withstand high temperatures.

Previous studies have shown that *T. scripta elegans* grows optimally at 26–30°C; however, incubation at 32°C results in only a 30% success rate, decreased liver catalase activity, and reduced *HSP70* mRNA expression compared to incubation at 28°C, suggesting that *T. scripta elegans* experience mild stress at 32°C (Gong et al. 2023; Wu 2018). Preliminary indoor experiments showed that *P. megacephalum* can tolerate 32°C for short periods, whereas higher temperatures are lethal. Thus, we selected 32°C as the heat stress condition and 25°C as the control to represent safe, stable conditions. In this study, we performed a comparative transcriptomic analysis of eight tissues (brain, heart, intestine, liver, lung, muscle, spleen, and stomach) under these conditions to: (1) identify differentially expressed genes (DEGs) between heat stress (32°C) and control (25°C) in the tissues of both turtle species; (2) analyze the functional categories of these DEGs; and (3) investigate the potential molecular mechanisms underlying the differences in heat tolerance between *P. megacephalum* and *T. scripta elegans*. This study could provide insights into our understanding of the molecular mechanisms underlying turtle responses to heat stress caused by climate change.

## 2 Materials and Methods

### 2.1 Sample Collection

Samples of 4‐year‐old female *P. megacephalum* (mean weight 249.4 ± 47.5 g) and female *T. scripta elegans* (mean weight 523.3 ± 53.5 g) were purchased from a breeding farm in Guangdong Province. Upon arrival, the turtles were acclimatized in an incubator at 25 ± 0.5°C for 2 weeks, with feeding every 2 days using turtle‐specific commercial feed (Shenzhen Cunjin, Guangdong, China). All experimental procedures in this study adhered to the ethical standards for animal experimentation established by the Guangdong Institute of Applied Biological Resource (GIABR20200501).

### 2.2 Heat Stress

Based on previous studies (Shen et al. 2013; Dang et al. 2019), a temperature of 32°C imposes significant heat stress on *P. megacephalum*, whereas in *T. scripta elegans*—despite 32°C representing the upper thermal limit for normal growth—it elicits measurable physiological responses without causing overt abnormalities. Given that the optimal temperature for both species is 25°C, the control group was maintained at 25°C, while the high‐temperature group was exposed to 32°C. This experimental design allows us to induce stress responses in both species while ensuring their survival. The turtles were housed in temperature‐controlled incubators (HWS‐150B, Tianjin Taisite), with a temperature variance of ±0.5°C. At the start of the experiment, three *P. megacephalum* and three *T. scripta elegans* were randomly selected and cultured at the control temperature of 25°C. The remaining turtles were placed in the experimental group, where the temperature was gradually increased from 25°C to 32°C at a rate of 1°C per hour. After maintaining the temperature at 32°C for 96 h, three individuals from each species were randomly selected for tissue sampling. Brain, heart, intestine, liver, lung, muscle, spleen, and stomach tissues were collected from six turtles in the experimental group and six turtles in the control group. The collected tissues were flash‐frozen in liquid nitrogen for at least 2 h and then stored in a −80°C freezer for RNA extraction.

### 2.3 Total RNA Extraction and Transcriptome Sequencing

RNA sequencing and bioinformatics analysis were conducted on 96 samples (2 species × 2 temperature groups × 8 tissues × 3 replicates). Total RNA was extracted using the Trizol RNA Isolation Kit (Sangon Biotech, Shanghai, China), following the manufacturer's instructions. The purity and quality of the total RNA were assessed using Nanodrop One. The sequencing libraries were prepared using the NEBNext Ultra RNA Library Prep Kit (NEB, USA). mRNA was enriched using magnetic beads with Oligo(dT), and double‐stranded cDNA was synthesized by reverse transcription using the enriched mRNA as a template. The double‐stranded cDNA was then purified, end‐repaired, A‐tailed, and ligated with sequencing adapters, followed by PCR amplification to construct the cDNA library (Parkhomchuk et al. 2009). After preliminary quantification and quality control of the cDNA libraries, sequencing was performed on the Illumina NovaSeq 6000 platform using a 150‐bp paired‐end strategy.

### 2.4 Data Processing

Raw data were quality‐filtered using Trimmomatic (Bolger et al. 2014) to remove adapters, contaminants, and low‐quality reads, discarding sequences shorter than 35 bp. The obtained clean data were aligned to the reference genomes (*P. megacephalum*: https://www.ncbi.nlm.nih.gov/datasets/genome/GCA_003942145.1/ and *T. scripta elegans*: https://www.ncbi.nlm.nih.gov/datasets/genome/GCF_013100865.1/) using HISAT2 (Kim et al. 2015). Then transcriptome assembly of the aligned reads was performed using StringTie (Pertea et al. 2015). Finally, functional annotation of the assembled transcripts was conducted using BLASTX, with annotations covering the NCBI non‐redundant (NR) protein database, Swiss‐Prot, Gene Ontology (GO), COG, KOG, Pfam, and Kyoto Encyclopedia of Genes and Genomes (KEGG) databases, setting an E‐value threshold of 1 × 10^−5^.

### 2.5 Evaluation of Replicate Correlation and Identification of DEGs

In transcriptome studies, biological replicates were included to reduce the impact of variability on gene expression results. To assess the correlation between replicate samples, the Pearson's correlation coefficient (*r*) was used. An *r* ^2^ closer to 1 indicates stronger correlation, reflecting higher reproducibility of the experiment (Schulze et al. 2012).

The fragments per kilobase of transcript per million mapped reads (FPKM) for each gene were calculated using StringTie (Florea et al. 2013) to determine relative gene expression levels. Differential expression analysis was performed using DESeq2 (Love et al. 2014) by comparing the FPKM values between the high‐temperature and control groups within each tissue of each species (e.g., heart at 25°C vs. 32°C) to identify DEGs. Genes with a fold change (FC) ≥ 1.5 or FC ≤ 2/3 and an adjusted *p* value (*q* value) < 0.01 were designated as DEGs.

Given the importance of chaperone‐related genes in adapting to high‐temperature environments (Shi et al. 2019; Xu et al. 2022; Jeyachandran et al. 2023), this study further analyzed the differential expression of these genes under high‐temperature conditions. By examining the DEGs in Excel files, chaperone‐related genes that exhibited expression under high temperature were identified. This analysis aimed to reveal potential differences in the molecular chaperone gene response between *P. megacephalum* and *T. scripta elegans* under heat stress and to explore the relationship between these differences and the species' heat tolerance.

### 2.6 The Enrichment GO and KEGG Pathways

To achieve standardized gene function classification, DEGs were mapped to GO terms using R/topGO (Alexa and Rahnenführer 2009), with a corrected *p* value (*q* value) < 0.05 set as the significance threshold for GO enrichment analysis. To further investigate the biological functions of these DEGs, KEGG pathway enrichment analysis was conducted. Significant pathways were identified using R/clusterProfiler (Yu et al. 2012), with a corrected *p* value (*q* value) < 0.05 used as the threshold for selecting significantly enriched pathways.

### 2.7 Protein–Protein Interaction Network Analysis to Identify Hub Genes

To identify hub genes, we conducted protein–protein interaction (PPI) network analysis following the enrichment of the protein processing in the endoplasmic reticulum pathway (ko04141) across multiple tissues (brain, heart, lung, and muscle). DEGs from these tissues, corresponding to proteins in this pathway, were entered into the STRING database (version 11.5) with a minimum interaction score of 0.400 (Szklarczyk et al. 2019). PPI networks were constructed and analyzed using the CytoHubba plugin in Cytoscape v3.10.3. The top 20 proteins identified by each of 10 topological methods (Degree, EPC, MNC, MCC, and six centrality measures: bottleneck, eccentricity, closeness, radiality, betweenness, and stress) were selected (Chin et al. 2014). The intersection of these top 20 proteins across all methods was used to define hub genes, which are predicted to play central roles in the protein processing pathway across the four tissues.

## 3 Results

### 3.1 Sequencing Data and Quality

After quality filtering, a total of 1 415 666 647 clean reads (∼424.03 Gb) were obtained from 48 *P. megacephalum* samples, with an average of 29 493 055 reads (∼8.83 Gb) per sample. On average, 86.85% (range: 72.80%–91.94%) of the clean reads were mapped to the *P. megacephalum* reference genome. For the 48 *T. scripta elegans* samples, a total of 1 357 010 445 clean reads (∼405.38 Gb) were obtained, with an average of 28 271 051 reads (∼8.45 Gb) per sample. On average, 81.38% (range: 70.68%–87.90%) of the clean reads were mapped to the *T. scripta elegans* reference genome (see Figure S1 and Table S1 for details).

### 3.2 Replicate Correlation and DEGs

The expression correlation heatmaps show that the correlation coefficients (*r* ^2^) between all biological replicates of both *P. megacephalum* and *T. scripta elegans* are generally above 0.7 (Figure S2), indicating high reproducibility. This confirms that the experimental design captured consistent gene expression patterns within each species, ensuring the reliability of the subsequent differential gene expression analysis.

**FIGURE 1:**
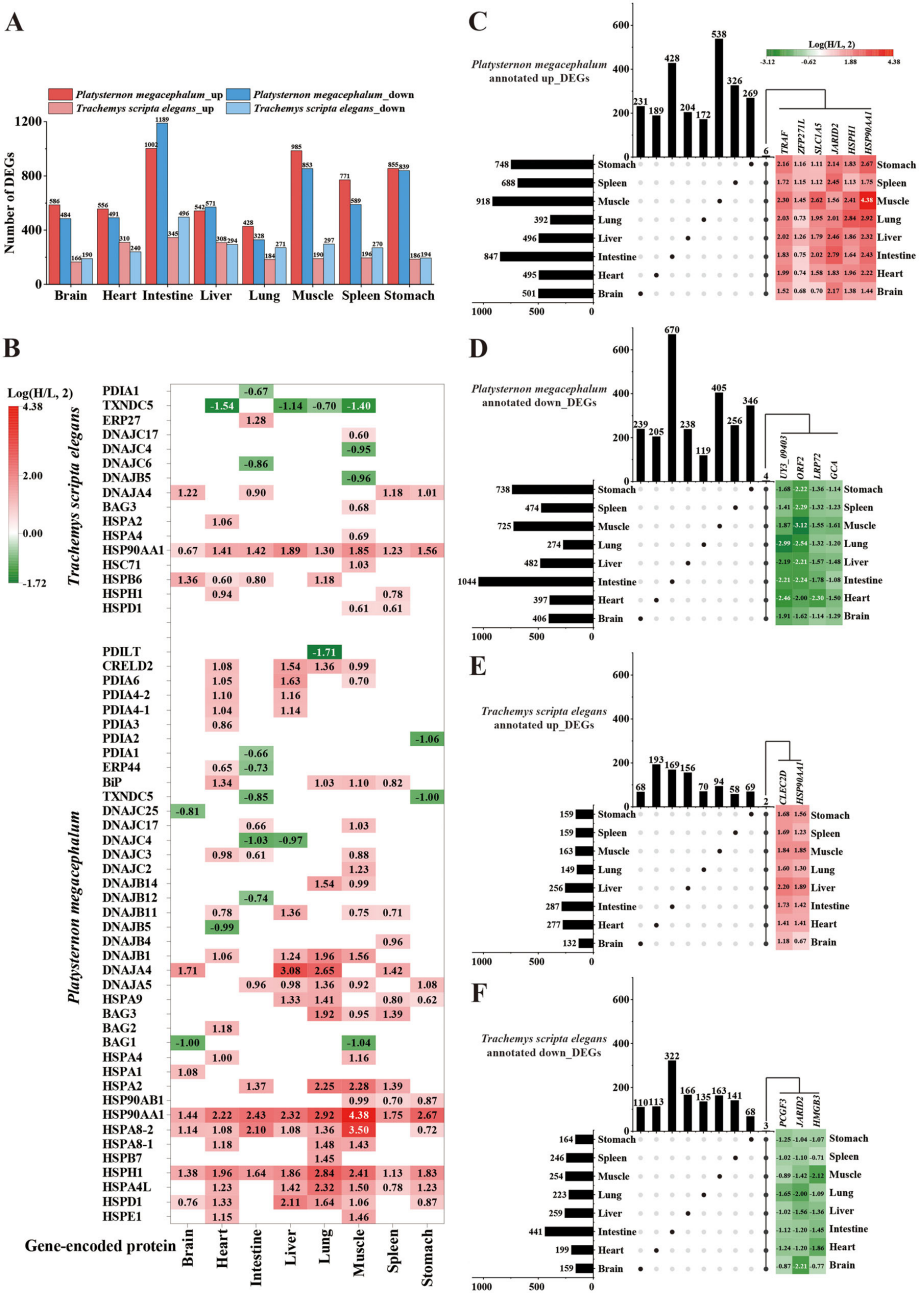
Statistical results of DEGs in different tissues and organs of P*latysternon megacephalum* and *Trachemys* *scripta elegans* under heat stress. (A) Number of DEGs in the two turtle species; (B) chaperone‐related DEGs; (C) up‐regulated DEGs annotated in *P. megacephalum*; (D) down‐regulated DEGs annotated in *P. megacephalum*; (E) up‐regulated DEGs annotated in *T. scripta elegans*; (F) down‐regulated DEGs annotated in *T. scripta elegans*. The intersections among the tissues and organs are shown in the middle section of (C)–(F) (with black dots indicating presence and gray dots indicating absence). The bar charts above (C)–(F) show the number of unique and shared DEGs for each tissue and organ, while the heatmaps on the right side of (C)–(F) display the DEGs shared among the tissues and organs.

For *P. megacephalum*, the number of up‐regulated genes exceeded the number of down‐regulated genes in the brain, heart, lung, muscle, spleen, and stomach, while the intestine and liver showed more down‐regulated than up‐regulated genes. In *T. scripta elegans*, the heart and liver exhibited more up‐regulated genes, whereas the brain, intestine, lung, muscle, spleen, and stomach had more down‐regulated genes. The number of DEGs in *P. megacephalum* ranged from 756 to 2191, in the order of intestine > muscle > stomach > spleen > liver > brain > heart > lung. For *T. scripta elegans*, the number of DEGs ranged from 356 to 851, in the order of intestine > liver > heart > muscle > spleen > lung > stomach > brain (Figure 1A). Overall, the number of DEGs in the eight tissues and organs of *P. megacephalum* was significantly higher than that in *T. scripta elegans*.

Annotation of the DEGs using seven databases revealed that approximately 86.85% and 84.55% of DEGs in the eight tissues of *P. megacephalum* and *T. scripta elegans*, respectively, were successfully annotated (Table S1). Ten DEGs were shared among the eight tissues and organs of *P. megacephalum*, including six up‐regulated genes (*HSP90AA1*, *HSPH1*, *TRAF*, *JARID2*, *SLC1A5*, *ZFP271L*) and four down‐regulated genes (*GCA*, *LRP72*, *ORF2*, *UY3_09403*), with the largest fold changes observed in muscle, heart, intestine, and lung (Figure 1C,D). In *T. scripta elegans*, five DEGs were shared among the eight tissues and organs, including two up‐regulated genes (*HSP90AA1*, *CLEC2D*) and three down‐regulated genes (*JARID2*, *HMGB3*, *PCGF3*) (Figure 1E,F and Table S2). Notably, *HSP90AA1* was up‐regulated in both species, but the up‐regulation in *P. megacephalum* was 1.2 to 2.4 times higher than in *T. scripta elegans*. Conversely, *JARID2* was up‐regulated in *P. megacephalum* but down‐regulated in *T. scripta elegans*. These results indicate that heat stress induces significant changes in gene expression levels in both species, with interspecies and inter‐tissue differences in DEG profiles and expression levels.

### 3.3 Differential Expression Analysis of Chaperone‐Related Genes

Analysis of the differential expression of chaperone‐related genes in various tissues and organs of *P. megacephalum* and *T. scripta elegans* revealed significant differences in the number and distribution of DEGs between the two species (Figure 1B and Table S2). A total of 40 DEGs were identified in the eight tissues and organs of *P. megacephalum*, with 70% being shared by at least two tissues. The genes for the heat shock proteins HSP 90‐α (*HSP90AA1*) and 105 kDa (*HSPH1*) were up‐regulated in all tissues, with the greatest up‐regulation of *HSP90AA1* observed in muscle tissue (log_2_(fold change) of 4.38). In contrast, the number of DEGs in *T. scripta elegans* was lower, with only 16 DEGs. *HSP90AA1* was up‐regulated in all tissues, with the highest up‐regulation observed in the liver (log_2_(fold change) of 1.89). These results indicate that the number of differentially expressed molecular chaperone‐related genes, and the degree of variation, are much higher in *P. megacephalum* than in *T. scripta elegans* under heat stress.

### 3.4 GO Functional Enrichment

**FIGURE 2:**
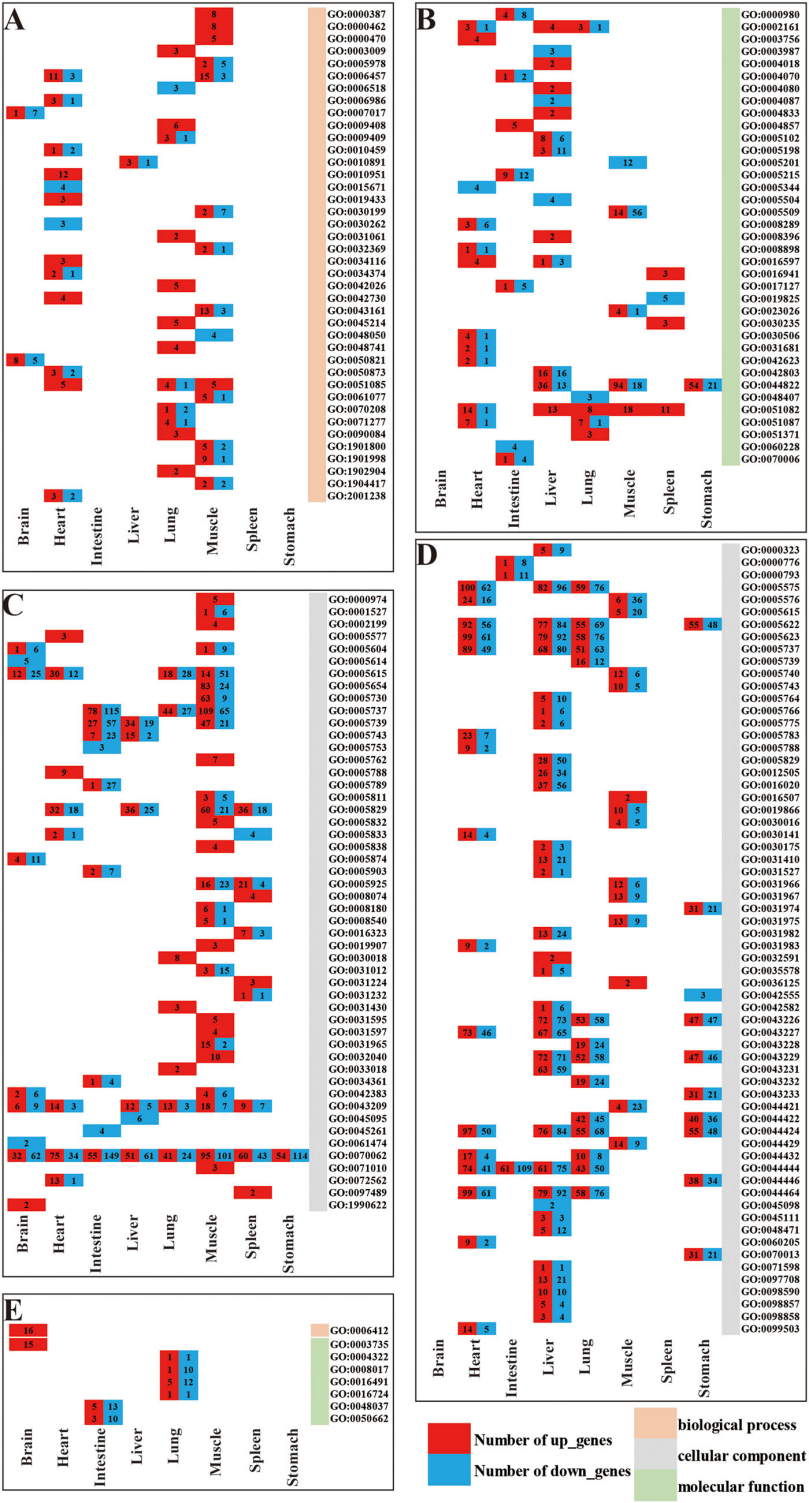
GO enrichment of DEGs in *Platysternon* *megacephalum* (A–C) and *Trachemys scripta elegans* (D,E) (*q* value < 0.05).

GO functional enrichment analysis revealed that 127 GO terms were enriched across the tissues and organs of *P. megacephalum*, including 40 biological process (BP) terms, 50 cellular component (CC) terms, and 37 molecular function (MF) terms. The muscle tissue of *P. megacephalum* had the highest number of enriched GO terms (BP:CC:MF = 14:27:5), followed by the heart tissue (BP:CC:MF = 13:8:11), with the stomach tissue having the fewest (BP:CC:MF = 0:1:1) (Figure 2 and Table S3). In contrast, the GO enrichment in the tissues and organs of *T. scripta elegans* was lower, with a total of 72 GO terms, of which 64 (88.9%) were related to cellular components, 1 to biological processes, and 7 to molecular functions (Figure 2). The liver tissue of *T. scripta elegans* had the most enriched GO terms (BP:CC:MF = 0:33:0), followed by the lung tissue (BP:CC:MF = 0:14:4), with no GO terms enriched in the spleen tissue. These results indicate that under heat stress, *P. megacephalum* enriched more GO terms than *T. scripta elegans*, with significant changes observed in BP, CC, and MF, reflecting its higher sensitivity to heat stress. In contrast, *T. scripta elegans* exhibited lower sensitivity, with changes primarily observed in CC.

### 3.5 KEGG Pathway Enrichment

**FIGURE 3:**
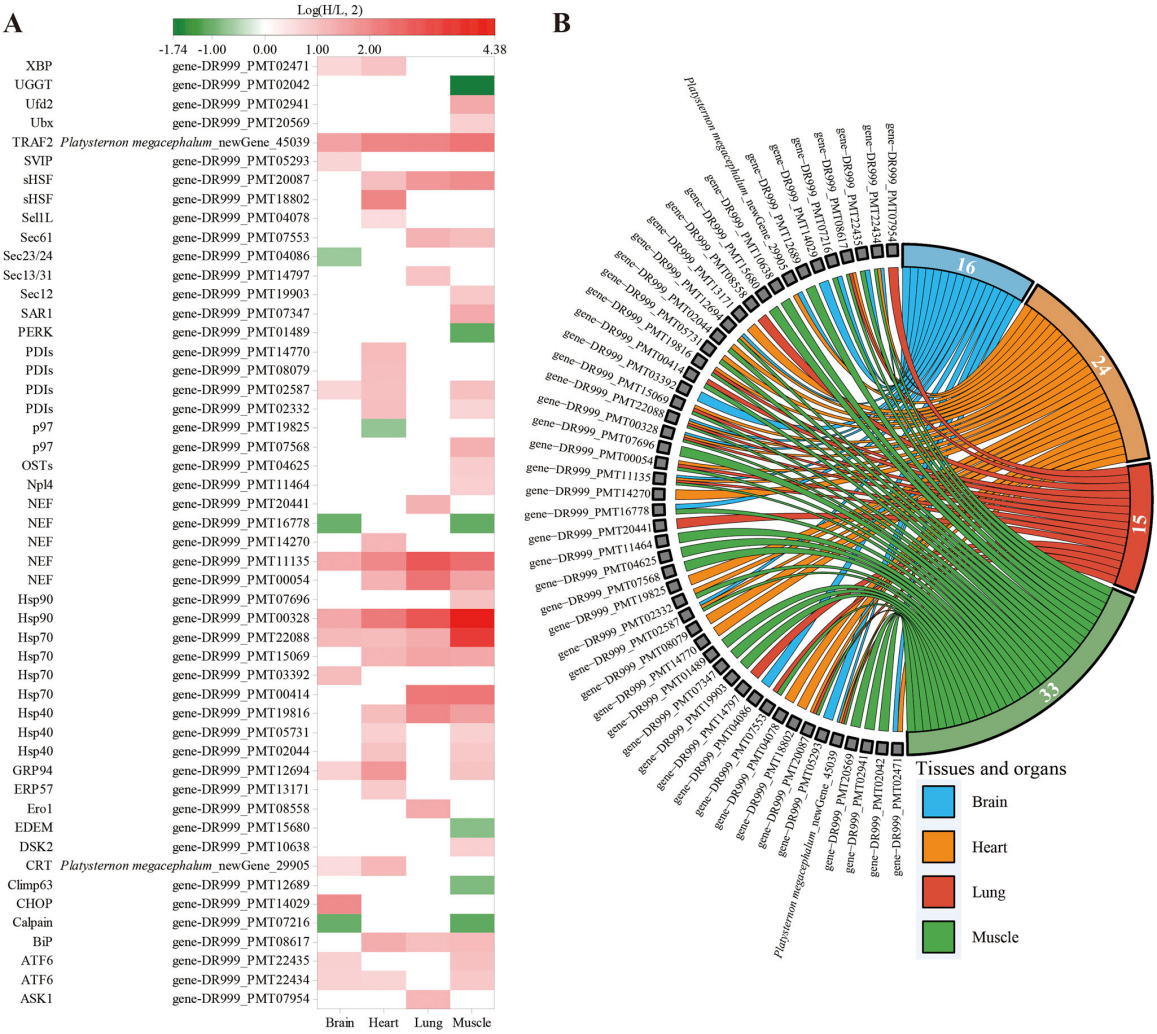
DEGs in the protein processing in endoplasmic reticulum pathway of *Platysternon* *megacephalum*. (A) Heatmap of DEGs in the brain, heart, lung, and muscle within the pathway: white indicates non‐DEGs. The second column represents gene IDs, and the first column shows the corresponding protein abbreviations. (B) DEGs in the pathway across significantly enriched tissues.

**FIGURE 4:**
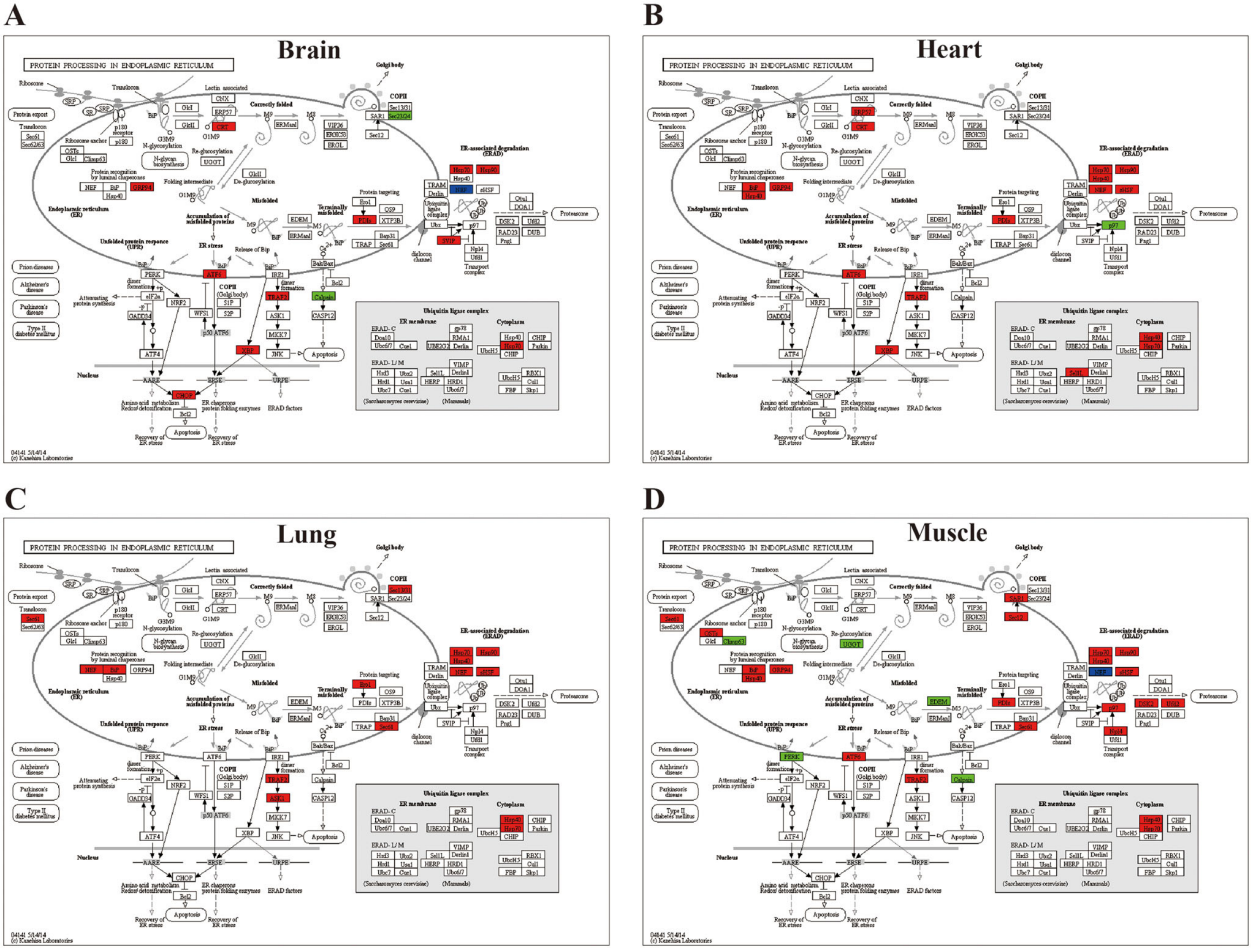
Enrichment of the protein processing in endoplasmic reticulum pathway in the brain (A), heart (B), lung (C), and muscle (D) of *Platysternon* *megacephalum*. (Red, green, and blue indicate genes with significantly up‐regulated, down‐regulated, and both up‐regulated and down‐regulated expression, respectively.)

KEGG enrichment analysis revealed that 18 metabolic pathways were significantly enriched across the eight tissues and organs of *P. megacephalum* (Table 1). Of these, 14 pathways were enriched in a single tissue or organ, with the intestine showing the highest number (6 pathways), primarily involving energy metabolism and amino acid synthesis, followed by heart and muscle with 2 and 3 pathways enriched, respectively. They involved carbohydrate and protein metabolism. The brain, spleen, and stomach each had one pathway enriched, which were involved in lipid metabolism and nitrogen metabolism. (Table 1; Figure S3 and Table S4). Additionally, four pathways were commonly enriched in two or more tissues and organs, including protein processing in endoplasmic reticulum, glutathione metabolism, biosynthesis of amino acids, and aminoacyl‐tRNA biosynthesis pathways (Table 1 and Figure S3). Notably, ko04141 protein processing in the endoplasmic reticulum pathway was enriched in the brain, heart, lung, and muscle, with significant up‐regulation of related genes, including *Hsp40*, *Hsp70*, *Hsp90*, *NEF*, and *ATF6* (Figures 3 and 4). Specifically, genes such as heat shock 70 kDa protein, molecular chaperone HtpG, heat shock protein 110 kDa, and TNF receptor‐associated factor 2 were up‐regulated in these tissues. Moreover, the ko00970 aminoacyl‐tRNA biosynthesis pathway was significantly enriched in the heart and liver of *P. megacephalum*, with overall gene expression up‐regulated. The ko03050 proteasome pathway and ko03008 ribosome biogenesis in eukaryotes pathways were significantly enriched in the muscle, with up‐regulated gene expression (Table 1 and Table S4). These results suggest that in response to heat stress, key tissues in *P. megacephalum* show significant up‐regulation of genes related to protein synthesis, which may indicate a compensatory response to heat stress, reflecting the considerable cellular stress faced under high temperatures. In terms of energy metabolism pathways, DEGs in *P. megacephalum* were significantly enriched in carbon metabolism, citrate cycle, and pyruvate metabolism pathways, with overall down‐regulation of gene expression, indicating that energy metabolism was inhibited under high temperatures, leading to adverse physiological responses that reduce tolerance to heat stress.

**TABLE 1:** KEGG enriched pathways in the tissues and organs of *Platysternon megacephalum* and *Trachemys scripta elegans*.

	*Platysternon megacephalum*	*q* value	*Trachemys scripta elegans*	*q* value
Brain	**ko04141 Protein processing in endoplasmic reticulum** ko00100 Steroid biosynthesis	0.0464 0.0464	ko03010 Ribosome	0.0000
Heart	**ko04141 Protein processing in endoplasmic reticulum** ko00980 Metabolism of xenobiotics by cytochrome P450 ko00480 Glutathione metabolism ko01230 Biosynthesis of amino acids ko00970 Aminoacyl‐tRNA biosynthesis ko00983 Drug metabolism—other enzymes	0.0000 0.0099 0.0099 0.0144 0.0298 0.0352	—	
Intestine	ko00620 Pyruvate metabolism ko00220 Arginine biosynthesis ko00020 Citrate cycle (TCA cycle) ko01200 Carbon metabolism ko01230 Biosynthesis of amino acids ko00630 Glyoxylate and dicarboxylate metabolism ko00480 Glutathione metabolism ko01210 2‐Oxocarboxylic acid metabolism	0.0009 0.0038 0.0046 0.0046 0.0065 0.0275 0.0385 0.0465	ko04110 Cell cycle	0.0000
Liver	ko00970 Aminoacyl‐tRNA biosynthesis	0.0196	—	
Lung	**ko04141 Protein processing in endoplasmic reticulum**	0.0182	—	
Muscle	**ko04141 Protein processing in endoplasmic reticulum** ko03050 Proteasome ko03008 Ribosome biogenesis in eukaryotes ko03040 Spliceosome	0.0005 0.0000 0.0147 0.0238	—	
Spleen	ko04520 Adherens junction	0.0352	—	
Stomach	ko00910 Nitrogen metabolism	0.0183	—	

*Note*: Pathways highlighted in bold represent the KEGG pathways most enriched across the tissues of *P. megacephalum*.

**FIGURE 5:**
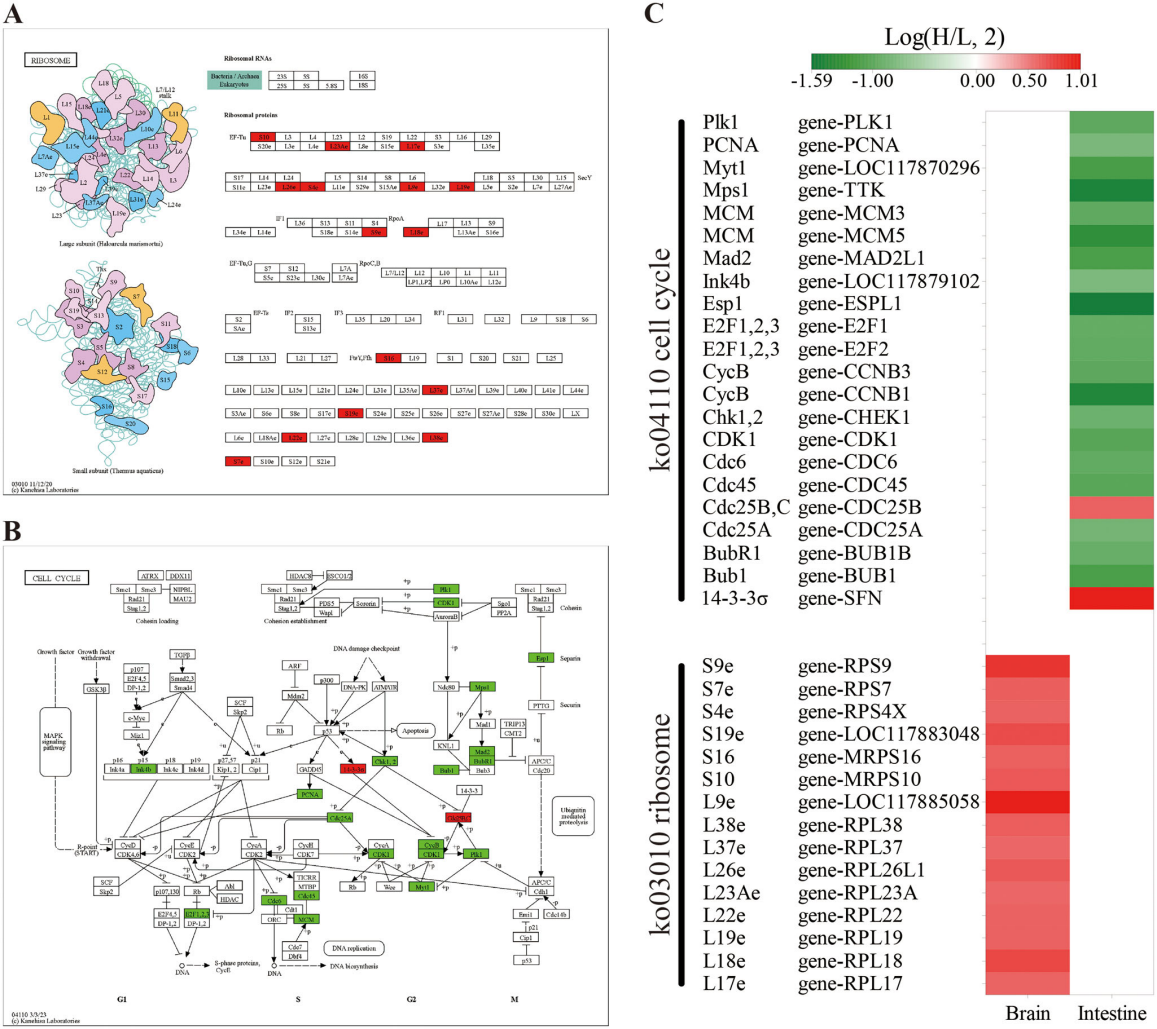
Enriched pathways in the brain and intestine of *Trachemys scripta elegans*. (A) Brain significantly enriched in the ribosome pathway. (B) Intestine significantly enriched in the cell cycle pathway; red and green indicate significantly up‐regulated and down‐regulated genes, respectively. (C) Heatmap of DEGs in the enriched pathways.

In comparison, the number of enriched pathways in *T. scripta elegans* was notably lower, with only two pathways identified, and no pathways shared across multiple tissues and organs. The enhanced pathways were the ribosome pathway in the brain and the cell cycle pathway in the intestine. In the ribosome pathway, all DEGs in the brain were up‐regulated, including genes such as *L17e*, *L18e*, *L23Ae*, *S10*, and *S9e* (Figure 5A,C). In the cell cycle pathway, except for gene‐SFN and gene‐CDC25B, all DEGs in the intestine of *T. scripta elegans* were down‐regulated (Figure 5B,C). The results showed that heat stress of 32°C had no significant effect on the tissue and organs of *T. scripta elegans*, which were subjected to a low degree of stress.

### 3.6 PPI Network Analysis to Identify Hub Genes

PPI network analysis was conducted to explore the protein processing in the endoplasmic reticulum pathway (ko04141) under heat stress in *P. megacephalum*. Fifty DEGs from four tissues (brain, heart, lung, and muscle) involved in this pathway were analyzed using the STRING database, resulting in a network of 50 nodes (four with no edges) and 319 edges (Figure S4 and Table S6).

**FIGURE 6:**
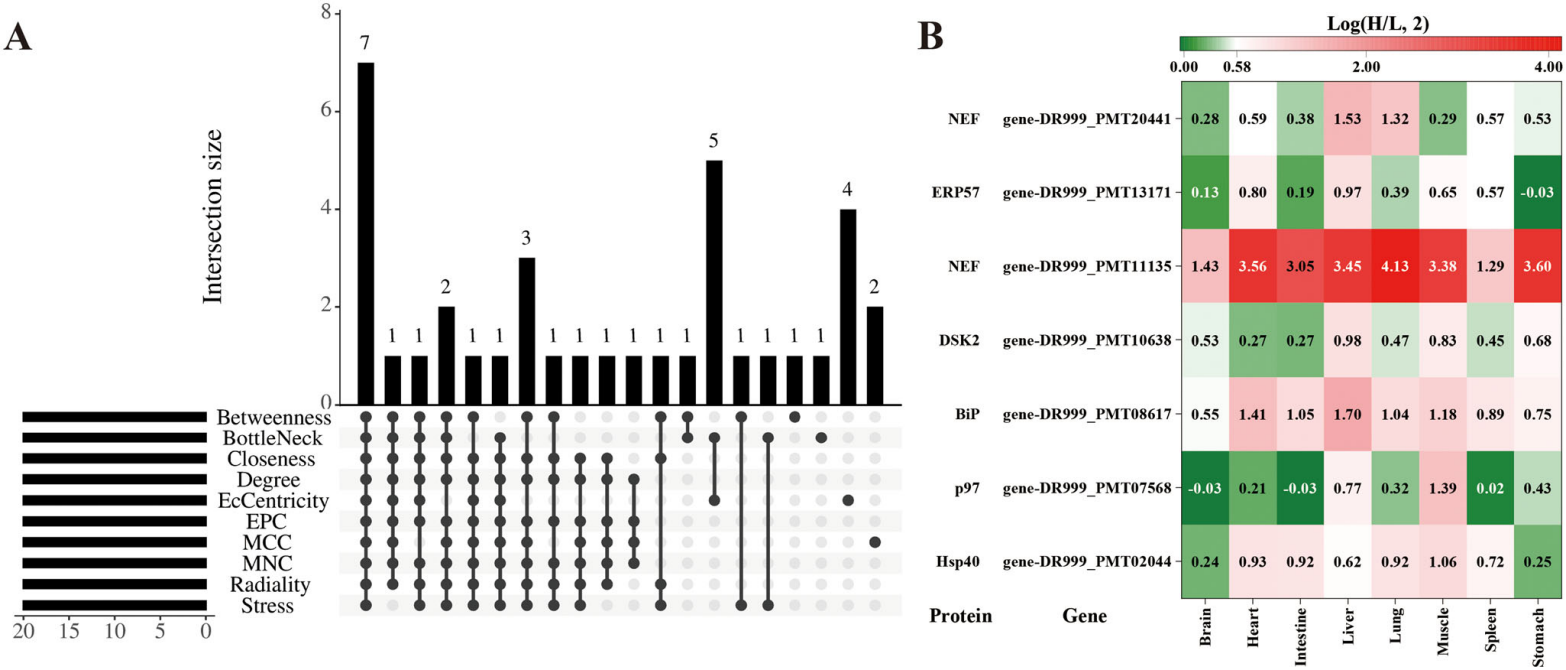
Identification of hub genes and their differential expression. (A) Upset plot illustrating the overlap of topological analysis methods across 10 distinct approaches. (B) Heatmap showing the fold change of hub gene expression in eight different tissues.

The top 20 proteins in the PPI network were identified using 10 topological methods. Seven proteins were consistently identified across all methods as key network components, corresponding to the genes: gene‐DR999_PMT02044 (*Hsp40*), gene‐DR999_PMT07568 (*p97*), gene‐DR999_PMT08617 (*BiP*), gene‐DR999_PMT10638 (*DSK2*), gene‐DR999_PMT11135 (*NEF*), gene‐DR999_PMT13171 (*ERP57*), and gene‐DR999_PMT20441 (*NEF*) (Figure 6 and Table S6). These genes were designated as hub genes. Nearly all hub genes were upregulated under heat stress, with gene‐DR999_PMT11135 (*NEF*) showing the most significant increase (log(H/L, 2) = 4.13). Additionally, gene‐DR999_PMT08617 (*BiP*) was significantly upregulated in all tissues except the brain, and gene‐DR999_PMT02044 (*Hsp40*) was upregulated in all tissues except the brain and stomach.

## 4 Discussion

### 4.1 Differential Effects of Heat Stress on Various Tissues

*P. megacephalum* exhibits pronounced, multi‐tissue, and multi‐pathway responses to heat stress, with distinct effects observed across different tissues (Table 1 and Table S4). In the brain, the activation of the ko04141 protein processing in endoplasmic reticulum pathway triggers the unfolded protein response (UPR), a process that may lead to apoptosis and neurodegeneration, while also compromising the brain's central role in thermoregulation and stress response, thereby severely endangering survival (Choi et al. 2010; Oyadomari and Mori 2004; Morrison and Nakamura 2019; Servili et al. 2020). In the intestine, the down‐regulation of energy metabolism pathways not only limits energy supply but may also impairs intestinal barrier function and increases permeability, potentially inducing systemic inflammatory responses (Tang et al. 2023; Zhou et al. 2024). In the liver, the enrichment of the ko00970 aminoacyl‐tRNA biosynthesis pathway and the up‐regulation of various tRNA synthetases (e.g., threonyl‐, leucyl‐, and glutamyl‐tRNA synthetases) indicate an attempt to repair heat‐induced damage through accelerated protein synthesis; however, this compensatory mechanism is energetically expensive and may exacerbate oxidative stress via increased reactive oxygen species (ROS) production (Schröder 2008). Moreover, in the spleen, the enrichment of the ko04520 adherens junction pathway, along with the up‐regulation of related genes (such as BAI1‐associated protein 2 and tight junction protein 1) and the down‐regulation of transcription factor 7, suggests compromised intercellular adhesion and signal transduction, potentially disrupting immune cell coordination (Li et al. 2022). In muscle tissue, the reorganization of four pathways—ko04141 (Protein processing in endoplasmic reticulum), ko03050 (Proteasome), ko03008 (Ribosome biogenesis in eukaryotes), and ko03040 (Spliceosome)—may lead to abnormal protein degradation and RNA splicing, thereby impairing muscle fiber stability and ultimately causing a significant decline in motor function (Rangan and Reck‐Peterson 2023; Hu et al. 2023; Zhang et al. 2022; Liu et al. 2023). Experimental observations reveal that after 12 h of incubation at 32°C, *P. megacephalum* exhibits symptoms of lethargy and anorexia. Overall, elevated temperatures disrupt proteostasis, interfere with energy metabolism, and impair intercellular signaling, collectively undermining the neural, immune, and motor functions of *P. megacephalum*.

In contrast, *T. scripta elegans* demonstrates a markedly milder response to heat stress. The number of DEGs in its tissues is substantially lower than that in *P. megacephalum*, with only limited pathway enrichment detected in the brain and intestine (Figure 1 and Table 1). In the brain, the up‐regulation of translation‐related genes (such as GO:0006412) (Figure 2) suggests that *T. scripta elegans* may mitigate heat‐induced stress by enhancing protein synthesis and folding, thereby preserving critical neural functions (Koo et al. 2015; Fan et al. 2024). In the intestine, the significant enrichment of the ko04110 cell cycle pathway, coupled with the overall down‐regulation of genes associated with cell cycle progression and checkpoint activation (Figure 5B,C), implies that the species might finely tune cell cycle and checkpoint mechanisms to reduce the energy expenditure associated with cell division and growth, thereby more effectively coping with heat stress (Kültz 2005). Furthermore, experimental observations indicate that under the same heat stress conditions, *T. scripta elegans* maintains normal levels of activity and feeding. Collectively, these tissue‐specific responses highlight the different thermal response mechanisms used by the two turtle species.

### 4.2 Key Genes Involved in the Response to Heat Stress

HSPs are essential for cellular stress responses, facilitating protein folding, preventing aggregation, and promoting degradation (Mayer and Bukau 2005). Previous studies have demonstrated up‐regulation of *HSP* genes in species such as *Ictalurus punctatus* (Xie et al. 2015) and *Eremias argus* (Chang et al. 2022) under heat stress, highlighting their crucial role in heat stress adaptation. In this study, *P. megacephalum* exhibited significantly higher expression of *HSP90AA1* and other *HSP* genes across all tissues compared to *T. scripta elegans*, with *HSP90AA1* expression being 1.2–2.4 times greater in *P. megacephalum* (Figure 1), suggesting a more sensitive heat stress response. In contrast, *T. scripta elegans* required higher temperatures to induce peak *HSP* expression in the liver and a higher threshold for *HSP* induction in muscle, consistent with findings by Zhang et al. (2023). While RNA‐seq accuracy is well‐established (Coenye 2021; Everaert et al. 2017), the absence of RT‐qPCR validation in this study presents a limitation. However, Zhang et al. (2023) reported similar trends in HSP‐related gene expression, supporting the findings of this study. Together, these findings underscore the critical role of HSPs in maintaining cellular stability under heat stress, while also highlighting that *T. scripta elegans* exhibits greater heat tolerance than *P. megacephalum*, with distinct species‐specific mechanisms of heat stress adaptation.

Additionally, this study showed that the jumonji and AT‐rich interaction domain containing 2 gene (*JARID2*) was up‐regulated in *P. megacephalum* and down‐regulated in *T. scripta elegans* under heat stress (Figure 1B,E). The JARID2 protein is associated with the Polycomb Repressive Complex 2 (PRC2), which can target and repress genes related to development, differentiation, and cell proliferation (Li et al. 2010; Landeira and Fisher 2011). Previous research has shown that overexpression of the *JARID2* gene in *Caretta caretta* embryos under high temperatures may disrupt brain cell proliferation and development (Bentley et al. 2017), highlighting its role in stress responses. Furthermore, in *Danio rerio*, the up‐regulation of *JARID2* inhibits the expression of key developmental and differentiation genes through the binding of the protein it encodes to PRC2, thus affecting cell pluripotency and self‐renewal capacity (Hanot et al. 2023). These studies suggest that changes in *JARID2* expression under heat stress may significantly impact development and adaptability. Specifically, the up‐regulation of *JARID2* in *P. megacephalum* under high temperatures may suppress genes related to cell proliferation and development, reducing cell proliferation and impairing the ability to repair heat‐induced damage. In contrast, the down‐regulation of *JARID2* in *T. scripta elegans* may reduce this suppression, enhancing the organism's ability to repair heat‐induced damage and improving heat tolerance. These findings provide new insights into how different species adjust the expression of key genes to cope with environmental stress. Additionally, other DEGs shared across the eight tissues, such as grancalcin isoform X4 (*GCA*) and leucine‐rich repeat‐containing protein 72 (*LRP72*), are relatively under studied for their function in thermotolerance and require further investigation.

The enrichment analysis of antioxidant genes revealed that under heat stress, antioxidant genes in *P. megacephalum* were enriched in GO terms related to the response to heat (GO:0009408), the response to unfolded proteins (GO:0006986), and protein disulfide isomerase activity (GO:0003756) (Figure 2). This suggests a pivotal role of these pathways in maintaining cellular homeostasis under stress. Several antioxidant genes, such as endoplasmic reticulum resident protein 44 and glutamate‐cysteine ligase (Table S5), were up‐regulated, indicating their potential involvement in mitigating oxidative damage during heat stress (Chen et al. 2021; Li et al. 2019b). In contrast, *T. scripta elegans* showed enrichment in GO terms like ferroxidase activity (GO:0004322), oxidoreductase activity (GO:0016491), and coenzyme binding (GO:0050662) (Figure 2), with the corresponding antioxidant genes, such as ceruloplasmin and peroxisomal acyl‐CoA oxidase 1, being generally down‐regulated (Table S5). This indicates a divergent strategy in dealing with oxidative stress, potentially involving the down‐regulation of antioxidant genes and the up‐regulation of other stress response mechanisms, similar to what has been observed in *Crassostrea virginica* under high temperatures (Rahman and Rahman 2021). The above analyses indicate both commonalities and differences in gene expression patterns between *P. megacephalum* and *T. scripta elegans* under heat stress. While both species exhibit consistent regulation of *HSP* gene expression, they differ in the expression of antioxidant genes and *JARID2*, and such differences may lead to differences in the adaptation of the two species of turtles to heat stress.

### 4.3 Regulatory Pathways Related to Heat Tolerance

*P. megacephalum* is typically found in cool, mountain stream environments and rarely encounters high temperatures under natural conditions (Shen et al. 2013). However, when exposed to acute heat stress, *P. megacephalum* significantly enriches the protein processing in the endoplasmic reticulum pathway in the brain, heart, lung, and muscle tissues (Figure 4). In contrast, the generalist *T. scripta elegans* (Burger 2009), which thrives in a broader range of habitats, likely exhibits a more flexible and robust ER stress response. This enhanced adaptability is probably a result of its regular exposure to temperature fluctuations, enabling it to better cope with thermal challenges (Huey et al. 2012). Recent studies of reptile ER stress have revealed similar regulatory mechanisms; For example, investigation in *Gallotia galloti* has shown that exposure to thermal fluctuations can trigger ER stress markers and activate the protective UPR (Gilbert et al. 2024; Yap et al. 2021). This ecological divergence suggests that the ability to cope with heat stress may differ significantly between specialists and generalists, shaping their adaptive responses (Richter‐Boix et al. 2015). Notably, our study is the first to identify and characterize this regulatory pathway (protein processing in endoplasmic reticulum) in turtles, thereby broadening our understanding of conserved cellular mechanisms for coping with heat stress across reptiles.

Heat stress can lead to protein misfolding and damage, causing accumulation in the ER and cytoplasm (Bánhegyi et al. 2007). The up‐regulation of genes related to the ER protein processing pathway in *P. megacephalum*, such as HSPs and crystallin αB (Figures 3 and 4), suggests that the UPR is activated as a protective mechanism. Notably, key regulators of the UPR—including activating transcription factor 6 (ATF6), X‐box binding protein 1 (XBP1), and TNF receptor‐associated factor 2 (TRAF2)—were significantly up‐regulated in the brain tissue under heat stress, promoting the expression of ER chaperones, foldases, ER‐associated degradation (ERAD), and autophagy‐related genes (Tepedelen and Kirmizibayrak 2019; Thuerauf et al. 2007). Moreover, ATF6 and XBP1 can bind to the cis‐acting elements of the DNA damage‐inducible transcript 3 (CHOP) gene, regulating its expression. CHOP promotes apoptosis by inhibiting the function of anti‐apoptotic family members such as BCL‐2 (Bánhegyi et al. 2007). This study also found that *CHOP* was up‐regulated in the brain tissue of *P. megacephalum* (Figure 4A), a critical step in ER stress‐induced apoptosis, where overexpression can lead to cell cycle arrest and/or apoptosis (Oyadomari and Mori 2004; Maytin et al. 2001; Zinszner et al. 1998). Thus, the up‐regulation of *CHOP* may weaken the heat tolerance of *P. megacephalum*, reducing its ability to adapt to heat stress.

Furthermore, studies have shown that under heat stress, the enrichment of steroid biosynthesis pathways and the down‐regulation of related genes in *Rhynchocypris oxycephalus* and *Danio rerio* may impair lipid metabolism, thereby reducing the cells’ ability to counteract ROS (Long et al. 2012; Yu et al. 2018; Juan et al. 2021; Sun et al. 2019). This study also found that steroid biosynthesis pathway genes were down‐regulated in the brain tissue of *P. megacephalum* under heat stress (Table S4), which may lead to weakened lipid metabolism and further reduce the species' ability to adapt to heat stress.

In contrast, *T. scripta elegans* exhibited limited KEGG pathway enrichment, with only the ribosome pathway in brain tissue and the cell cycle pathway in intestinal tissue (Figure 5). Studies suggest that up‐regulating ribosome‐related genes under heat stress enhances protein synthesis and corrects protein misfolding, supporting intracellular protein homeostasis (Li et al. 2021; Knapp and Huang 2022). For example, in *Megalobrama amblycephala* liver, up‐regulation of ribosome pathway genes helped mitigate heat‐induced protein damage (Li et al. 2019a), and in *Oncorhynchus mykiss*, similar up‐regulation improved protein folding under heat stress (Zhao et al. 2024). The significant up‐regulation of ribosome pathway genes in *T. scripta elegans* brain tissue under heat stress (Figure 5A,C) suggests that this response may help the species better cope with high temperatures by enhancing protein synthesis and folding.

Conversely, the down‐regulation of cell cycle pathway genes in the intestinal tissue of *T. scripta elegans* under heat stress, including genes related to cell cycle and checkpoint activation (Figure 5B,C), reflects a common strategy to reduce energy consumption associated with cell division and growth. By down‐regulating these genes, organisms conserve energy for stress response mechanisms, mitigating the damage caused by heat stress (Kültz 2005). For example, Jesus et al. (2016) found that high temperatures led to the down‐regulation of cell cycle‐related genes in *Squalius torgalensis*, inhibiting growth processes and redirecting energy from growth to stress response mechanisms to cope with the adverse effects of heat stress. Similar results were validated by Buckley et al. (2006), showing that organisms typically reduce metabolic activity by inhibiting cell cycle gene expression, prioritizing energy allocation to counteract environmental stress (López‐Maury et al. 2008). These studies suggest that *T. scripta elegans* may similarly down‐regulate cell cycle‐related genes to prioritize energy for repairing damaged molecules (e.g., proteins and membranes), helping to mitigate heat‐induced cellular damage and adapt to high temperatures.

## 5 Conclusion

Under heat stress, *P. megacephalum* and *T. scripta elegans* exhibited both common and distinct molecular response mechanisms. Both species showed significant differential expression of heat shock protein genes. *P. megacephalum* exhibited a stronger stress response in muscle, heart, brain, and intestinal tissues, particularly with the significant up‐regulation of the protein processing in endoplasmic reticulum pathway and the *CHOP* gene in brain. Additionally, genes related to energy metabolism and lipid metabolism were down‐regulated, while the *JARID2* gene, which inhibits cell proliferation and development, was up‐regulated. Within protein processing in endoplasmic reticulum pathway, seven hub genes were identified in *P. megacephalum*, namely gene‐DR999_PMT02044 (*Hsp40*), gene‐DR999_PMT07568 (*p97*), gene‐DR999_PMT08617 (*BiP*), gene‐DR999_PMT10638 (*DSK2*), gene‐DR999_PMT11135 (*NEF*), gene‐DR999_PMT13171 (*ERP57*), and gene‐DR999_PMT20441 (*NEF*). These molecular responses may limit *P. megacephalum*’s ability to adapt to heat stress. In contrast, *T. scripta elegans* showed up‐regulation of ribosome pathway genes and down‐regulation of cell cycle pathway genes, enhancing protein synthesis and cellular repair capabilities. Furthermore, down‐regulation of *JARID2* in *T. scripta elegans* supported normal cell development and proliferation, improving heat stress adaptability. This study is the first to highlight the critical role of protein processing in the endoplasmic reticulum pathway in turtle heat stress responses, offering new insights into their molecular mechanisms and adaptation potential to future climate warming.

## Conflicts of Interest

The authors declare no conflicts of interest.

## Supporting information




**Figure S1** Comparison of reads mapped to the reference genome in *Platysternon megacephalum* and *Trachemys scripta elegans*.
**Figure S2** Heatmap of expression correlation between samples of *Platysternon megacephalum* (A) and *Trachemys scripta elegans* (B).
**Figure S3** KEGG pathways enriched in various tissues and organs of *Platysternon megacephalum* and *Trachemys scripta elegans* (Pathways enriched in more than one tissue or organ are highlighted in red.)
**Figure S4** Protein‐protein interaction (PPI) network.

**Table S1** Sequencing data, quality statistics and annotation of DEGs

**Table S2** DEGs shared by both turtle tissues, molecular chaperone‐related genes

**Table S3** GO‐enriched terms for DEGs

**Table S4** Other significantly enriched pathways

**Table S5** Antioxidant‐related GO enrichment terms

**Table S6** Protein‐protein interaction network analysis to identify hub gene profiles
