# Supplementary material for: Comparative Analysis of Heat Exposure‐Induced Molecular Changes in Two Turtle Species with Contrasting Thermal Adaptations
Source: Integr Zool. 2025 Jul 17;21(3):609–23. doi: 10.1111/1749-4877.13011 (PMC13165693; doi:10.1111/1749-4877.13011)
Supplement: Supplementary file 1 — Figure S1 Comparison of reads mapped to the reference genome in Platysternon megacephalum and Trachemys scripta elegans. Figure S2 Heatmap of expression correlation between samples of Platysternon megacephalum (A) and Trachemys scripta elegans (B). Figure S3 KEGG pathways enriched in various tissues and organs of Platysternon megacephalum and Trachemys scripta elegans (Pathways enriched in more than one tissue or organ are highlighted in red.) Figure S4 Protein‐protein interaction (PPI) network. [file INZ2-21-609-s002.docx]

**Figure S1** Comparison of reads mapped to the reference genome in *Platysternon megacephalum* and *Trachemys scripta elegans.*

(H and L represent samples from the high-temperature treatment group and control group of *P. megacephalum*, respectively. HHE and LHE represent samples from the high-temperature treatment group and control group of *T. scripta elegans*, respectively. The abbreviations bra, hea, int, liv, lun, mus, spl, and sto correspond to the brain, heart, intestine, liver, lung, muscle, spleen, and stomach samples from the turtles.)

**Figure S2** Heatmap of expression correlation between samples of *Platysternon megacephalum* (A) and *Trachemys scripta elegans* (B).

(The closer r^2^ is to 1, the stronger the correlation between the two replicates. H and L represent samples from the high-temperature treatment group and control group of *P. megacephalum*, respectively. HHE and LHE represent samples from the high-temperature treatment group and control group of *T. scripta elegans*, respectively. The abbreviations bra, hea, int, liv, lun, mus, spl, and sto correspond to the brain, heart, intestine, liver, lung, muscle, spleen, and stomach samples from the turtles.)


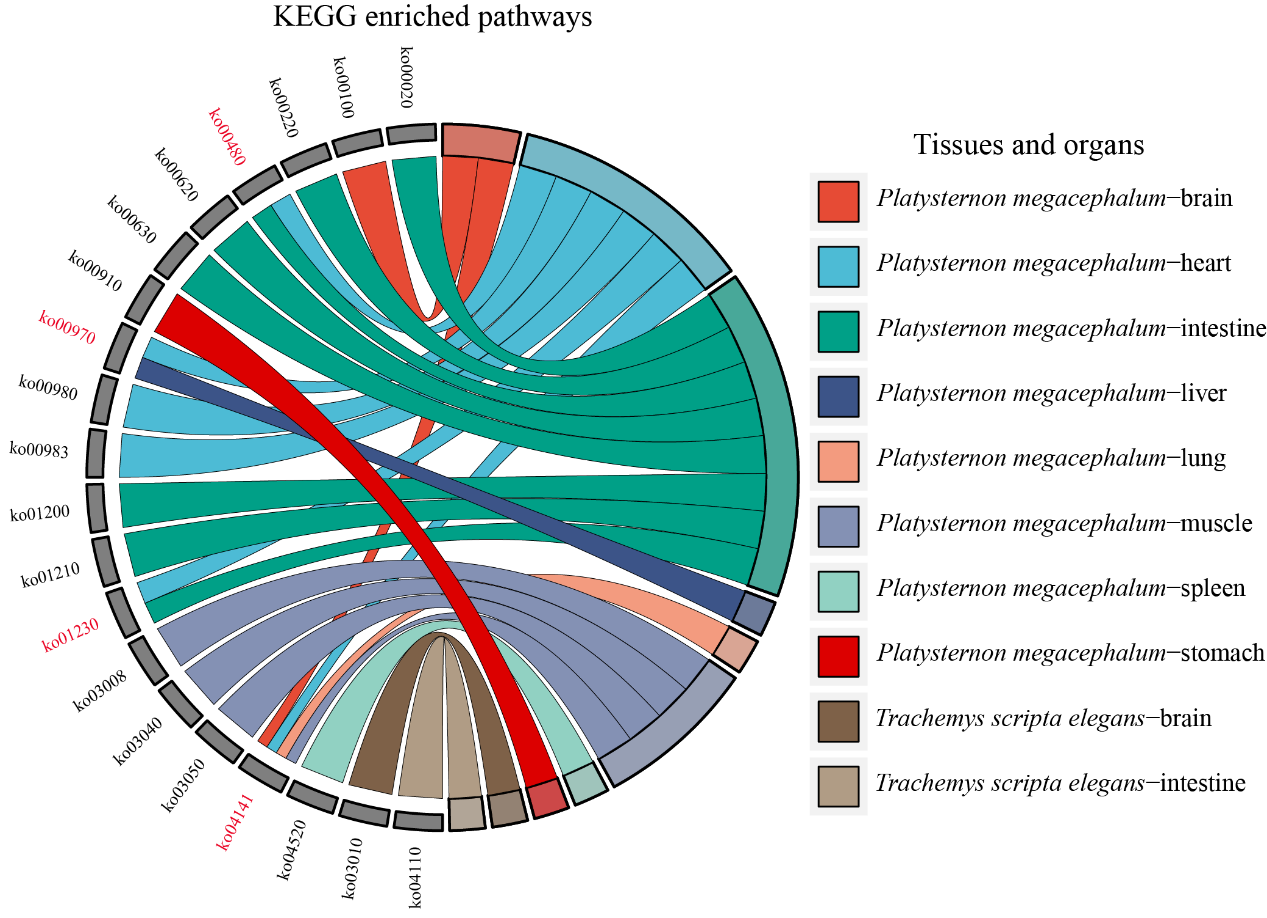


**Figure S3** KEGG pathways enriched in various tissues and organs of *Platysternon megacephalum* and *Trachemys scripta elegans* (Pathways enriched in more than one tissue or organ are highlighted in red.)


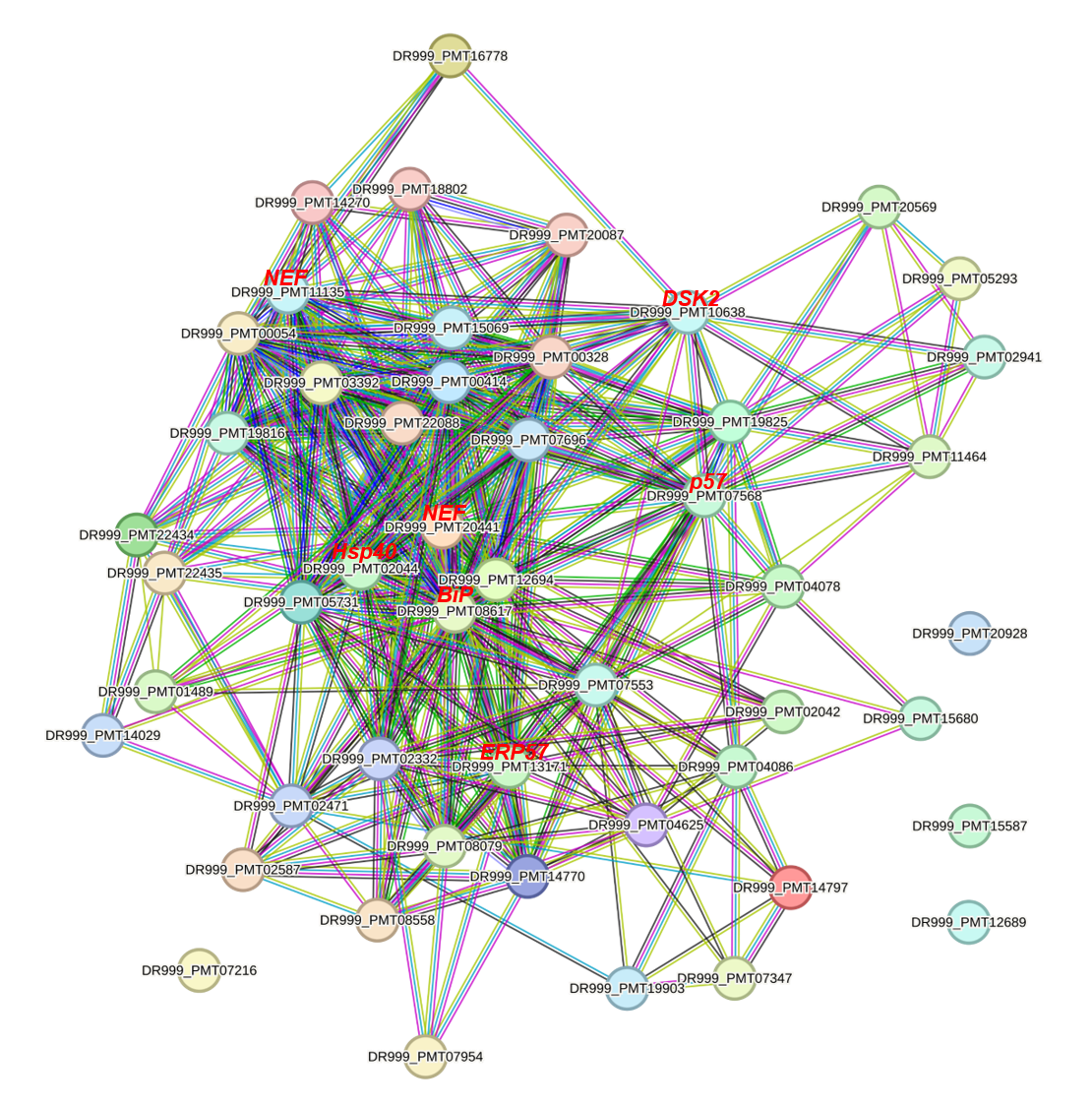


**Figure S4** Protein-protein interaction (PPI) network.

(The network consists of 50 nodes and 319 edges, with an average node degree of 12.8 and an average local clustering coefficient of 0.599. The expected number of edges is 40, and the PPI enrichment analysis yielded a p-value of < 1.0e-16. The part of the figure marked in red is the hub gene obtained by using ten topological methods.)
